# Retraction: Metal-ion batteries for electric vehicles: current state of the technology, issues and future perspectives

**DOI:** 10.1039/d4na90052a

**Published:** 2024-04-29

**Authors:** Jaya Verma, Deepak Kumar

**Affiliations:** a Centre for Automotive Research and Tribology (CART), Indian Institute of Technology Delhi New Delhi 110016 India jayaverma@iitd.ac.in

## Abstract

Retraction of ‘Metal-ion batteries for electric vehicles: current state of the technology, issues and future perspectives' by Jaya Verma *et al.*, *Nanoscale Adv.*, 2021, **3**, 3384–3394, https://doi.org/10.1039/D1NA00214G.

The Royal Society of Chemistry, with the agreement of the authors, hereby wholly retracts this *Nanoscale Advances* review article due to significant portions of text overlap with a number of sources throughout the review article, in particular ref. 16, 38, 39 and 47 of the article and ref. 1–4 below, which were not cited. Although many of these articles have been cited and the source material referenced, it was not made clear that significant sections of the text were reproduced from these articles.

Signed: Jaya Verma and Deepak Kumar

Date: 24/04/2024

Retraction endorsed by Jeremy Allen, Executive Editor, *Nanoscale Advances*

## Supplementary Material
